# Polycaprolactone/graphene oxide/acellular matrix nanofibrous scaffolds with antioxidant and promyelinating features for the treatment of peripheral demyelinating diseases

**DOI:** 10.1007/s10856-023-06750-2

**Published:** 2023-10-05

**Authors:** Aishwarya Nagarajan, Nasera Rizwana, Michelle Abraham, Mahima Bhat, Aakanksha Vetekar, Goutam Thakur, Uttara Chakraborty, Vipul Agarwal, Manasa Nune

**Affiliations:** 1 Manipal Institute of Regenerative Medicine, Bengaluru, Manipal Academy of Higher Education, Manipal, 576104 Karnataka India; 2 Department. of Biomedical Engineering, Manipal Institute of Technology, Manipal Academy of Higher Education, Manipal, 576104 Karnataka India; 3 Cluster for Advanced Macromolecular Design (CAMD), School of Chemical Engineering, University of New South Wales, Sydney, NSW 2052 Australia

**Keywords:** Peripheral demyelinating diseases, Schwann cells, graphene oxide, polycaprolactone, electrospun scaffolds, acellular matrix

## Abstract

Peripheral demyelinating diseases entail damage to axons and Schwann cells in the peripheral nervous system. Because of poor prognosis and lack of a cure, this group of diseases has a global impact. The primary underlying cause of these diseases involves the inability of Schwann cells to remyelinate the damaged insulating myelin around axons, resulting in neuronal death over time. In the past decade, extensive research has been directed in the direction of Schwann cells focusing on their physiological and neuroprotective effects on the neurons in the peripheral nervous system. One cause of dysregulation in the remyelinating function of Schwann cells has been associated with oxidative stress. Tissue-engineered biodegradable scaffolds that can stimulate remyelination response in Schwann cells have been proposed as a potential treatment strategy for peripheral demyelinating diseases. However, strategies developed to date primarily focussed on either remyelination or oxidative stress in isolation. Here, we have developed a multifunctional nanofibrous scaffold with material and biochemical cues to tackle both remyelination and oxidative stress in one matrix. We developed a nanofibrous scaffold using polycaprolactone (PCL) as a foundation loaded with antioxidant graphene oxide (GO) and coated this bioscaffold with Schwann cell acellular matrix. In vitro studies revealed both antioxidant and remyelination properties of the developed bioscaffold. Based on the results, the developed multifunctional bioscaffold approach can be a promising biomaterial approach for treating demyelinating diseases.

**Graphical Abstract:**

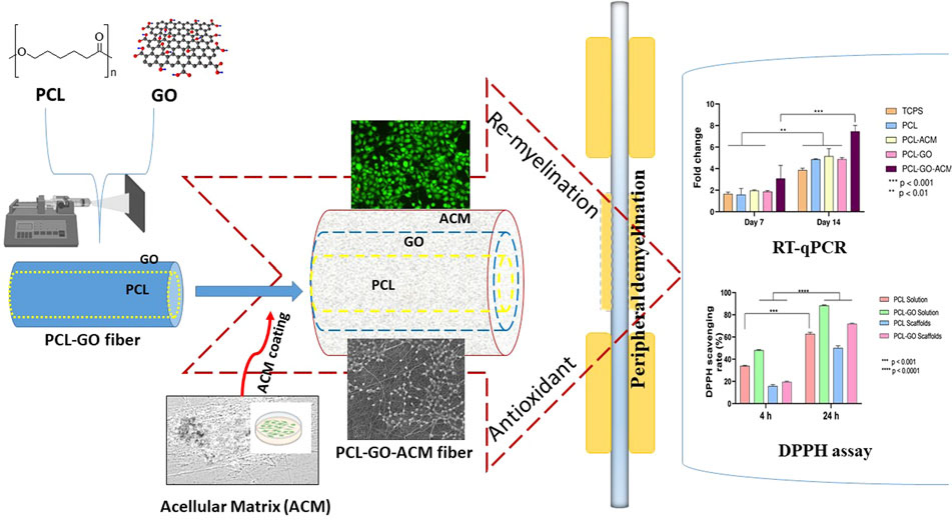

## Introduction

Peripheral demyelinating diseases are a group that includes diseases like Guillain-Barre syndrome, chronic inflammatory demyelinating polyradiculoneuropathy, anti-myelin associated glycoprotein neuropathy, and Charcot Marie tooth disease [1]. These diseases involve damage to the axons and glial cells specifically Schwann cells in the peripheral nervous system which is responsible for myelination [2, 3]. Injury to myelin sheath or demyelination can occur due to various factors like infections, genetic mutations, and auto-immune diseases. The most common risk factor for diseases involving demyelination is oxidative stress [4, 5]. In conditions such as inflammation, there is an increase in free radicals that can overcome the antioxidant defenses within the cells and thereby leading to increased stress leading to mitochondrial damage causing cell death and demyelination [6]. One of the important features of inflammatory demyelinating diseases is reactive oxygen species (ROS) production which has adequate evidence from previous studies [7–10].

To reduce the levels of free radicals and also improve myelination, various treatment strategies like cell-based therapeutic approaches, human bone marrow-derived stem cells, and natural products have been employed [1, 11–14]. Nanocomposites have also been successfully utilized for the treatment of demyelinating diseases such as multiple sclerosis. A natural antioxidant in a form of a nanodroplet formulation of pomegranate seed oil (nano-PSO) has been shown to significantly reduce oxidative stress and demyelination in multiple sclerosis-induced mice [15]. However, some limitations exist such as biocompatibility issues, site-specific targeting, and improper blood brain barrier penetration [16].

Although the peripheral nervous system has regeneration ability, it is limited to small injuries and the complex nature of the Schwann cell morphology and electrical activity demands effective strategies for regeneration [17–19]. The application of nanomaterials and nanocomposite scaffolds play an important role in the sprouting of axons [20], and activating signaling pathways for neural regeneration [21]. Electrospun scaffolds such as poly-L-lactic acid, poly(ε-caprolactone) (PCL), and polyethylene glycol have been studied very extensively as these provide guidance cues for cell proliferation and differentiation [22–26]. However, they lack the biochemical cues which is required to reduce the free radicals and also provide a microenvironment for the glia to improve myelination and nerve regeneration [21, 27].

Graphene oxide (GO) is a derivative of single-layer graphene synthesized by oxidation of graphene followed by ultrasonic exfoliation by Modified Hummer’s method [28]. It consists of various functional groups such as hydroxyl, carboxyl and epoxide groups on its surface facilitating its dispersion in water. The hydroxyl and epoxide functional groups provide reactive oxygen sites which can be functionalized with other molecules by hydrogen bonding, van der Waals forces and covalent bonding. The carboxyl groups that are present at the edges of GO sheets are responsible for its dispersion in water [29–31]. The structure, size, functional groups and π-π* stacking interactions of GO enables it to be used in drug delivery, cell attachment, proliferation and cell differentiation [32–34].

Acellular matrix (ACM) is a complex, three-dimensional highly organized structure which is devoid of cells and comprises fibrous proteins and glycosaminoglycan-based components [35–37]. It provides mechanical support and adhesion receptors for cell adhesion, proliferation and differentiation. Acellular matrix from various sources of mesenchymal stem cells have reduced the oxidative stress efficiently [38, 39]. In peripheral nerve regeneration, Schwann cells are known to produce growth factors like nerve growth factors, brain derived growth factor, which are known to help in axon regeneration and myelination. In addition, interactions of Schwann cells and basal lamina of the Schwann cell acellular matrix have a role in promoting myelination [40]. We have previously demonstrated that Schwann cells also secrete extracellular matrix which helped to provide biochemical cues to improve cell proliferation and adhesion on the PCL nanofibrous scaffolds [24]. In another work, we also confirmed that Schwann cell acellular matrix when coated on PCL and multiwalled carbon nanotube scaffolds showed a significant expression of peripheral myelin protein 22 (PMP22) gene [41]. Based on these results, we deduced that Schwann cell matrix in combination with PCL scaffolds could promote more functional neuronal properties such as myelination.

To address the complex neurological disorders such as demyelination and oxidative damage, it is ideal to have a multifunctional approach. Previous studies so far have focussed on either of the two issues in isolation i.e. demyelination or oxidative damage but no attempts have been made to address both these factors (demyelination and oxidative damage) in one study using a multimodal scaffold. Here, we fabricated multimodal PCL-GO nanofibrous scaffolds and subsequently also coated them with Schwann cell derived acellular matrix (PCL-GO-ACM) where GO is included for its potential antioxidant properties while Schwann cell derived acellular matrix will provide the microenvironment and biochemical cues to promote cell adhesion, proliferation and remyelination. Using multiple biochemical techniques, we observed both antioxidative and pro-myelinating effects of fabricated PCL-GO and PCL-GO-ACM in vitro.

## Materials and methods

### Materials

Polycaprolactone (PCL, *MW* 80,000 g/mol, Cas no. 440744) and Phosphate buffer saline tablets (P4417) was purchased from Sigma-Aldrich (India). Methanol (AS058), chloroform (MB109), and dimethyl sulfoxide (MB058) were purchased from HiMedia Laboratories Pvt Ltd (India). Rat Schwann cells (RSC-96) were obtained from American Type Culture Collection (CRL-2765 ATCC, USA). Fetal bovine serum (FBS) (lot no 2440096), antibiotics penicillin-streptomycin (P4333), antibiotic-antimycotic (A5955), and Dulbecco’s eagle medium (DMEM) (lot no. 2323533) were purchased from Gibco (India). Cytochemical stains (alcian blue (CT-102), Masson’s trichrome, picrosirius red (ST-141), safranin O) were purchased from AMD Labs (India). Coomassie blue, methylene blue, MTT kit (TC191-1G), Triton-X (MB031), tween-20 (MB067) and DAPI (4’,6 Diamidino-2-phenylindole dihydrochloride) (MB097-10MG) were purchased from HiMedia Laboratories Pvt Ltd (India). Live/dead kit (lot no. 2015555), rhodamine-phalloidin (lot no. 2157163), secondary antibody (Alexa Fluor 488 rabbit anti-mouse) (Cat # A11059), anti-α-tubulin (bovine) (lot no WG3334405), mouse IgG1, monoclonal (Cat # A11126) were purchased from Invitrogen (USA). Graphene oxide (GO) was prepared by Hummer’s method [42].

### Preparation of PCL-GO solution

**Fig. 1:**
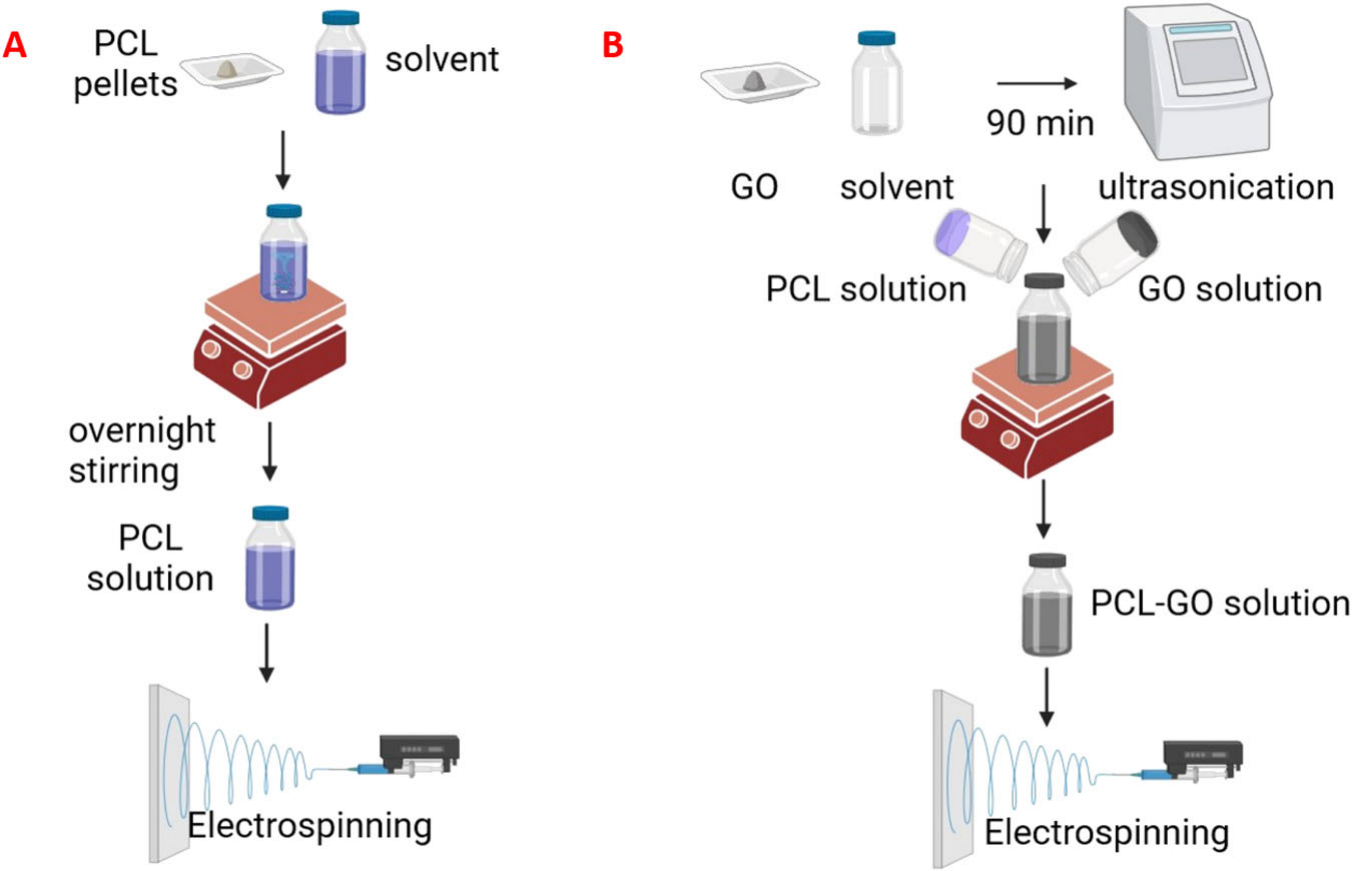
A schematic representing methodology of (**A**) electrospinning of PCL nanofibers and (**B**) electrospinning of PCL-GO nanofibers

GO (6 mg) was added to 1 ml of DMF and then sonicated in a water bath sonicator for 90 min in a closed bottle. Thereafter, 0.6 mg of PCL pellets of average molecular weight of 80,000 g/mol were dissolved in 5 ml of dichloromethane by mechanical stirring overnight at room temperature. Next, dissolved PCL solution was mixed with GO dispersion and homogenized by mechanical stirring for 5 h (Fig. 1).

### Fabrication of PCL-GO electrospun scaffolds

The nanofibrous scaffolds were fabricated with an electrospinning instrument HO-NFES-040 (Holmarc Opto-Mechatronics Ltd, Kochi, India) [43]. Next, PCL solution and PCL-GO dispersion were placed in a 15 ml glass syringe connected to a 20 G needle. To obtain random fibers, a flat stationary collector was attached to the system. The electrospinning conditions were fixed as; voltage of 16 kV, flow rate of 6 μl/min, and distance between the needle tip and stationary collector was kept at 20 cm. The electrospinning process was stopped after 10 ml of solution was ejected by gradually reducing the voltage to 0 kV. The obtained nanofibrous scaffolds were cut into circular shape of 1 mm diameter. The prepared nanofibrous scaffolds were stored in a vacuum desiccator before use.

### Cell line

Rat Schwann cells (RSC-96) were cultured in DMEM supplemented with 10% FBS, 1% penicillin/streptomycin and 1% antibiotic-antimycotic and incubated at 37 °C in 5% CO_2_ atmosphere. Culture media was changed every alternate day.

### Preparation of acellular matrix (ACM)

**Fig. 2:**
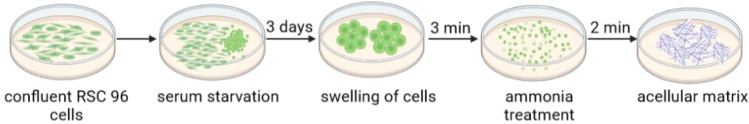
A schematic representing methodology of preparation of acellular matrix

After 80–100% confluency of RSC-96 cells was achieved, spent media was removed and discarded. Confluent cells were washed with PBS and subjected to serum free condition by adding DMEM without serum for 3–5 days [24]. Later, serum free media was removed and cells were washed once with PBS. Following which cells were incubated in Milli-Q (MQ) deionized water for 2–3 min to loosen the cell attachment with the plastic surface of the cultured wells. After removing MQ water, 0.1% ammonia solution was added to cells for 1–2 min at room temperature. The ammonia solution with the cells was then removed gently by inverting the plates. Any remaining cell debris was removed by giving three PBS washes. The obtained decellularized matrix was allowed to dry overnight in the laminar flow cabinet, washed thrice with PBS and scrapped using cell scrapper. The obtained ACM dispersed in in PBS was supplemented with penicillin/streptomycin (1%) and stored at −80 °C for future use (Fig. 2).

### Fabrication of ACM coated neat PCL and PCL-GO nanofibrous scaffold

The PCL and PCL-GO scaffolds were deposited on circular glass coverslips. These scaffolds on coverslips were UV sterilized for 1 h. The PCL-GO circular coverslips were placed on the vacuum chuck of the spin coating machine HO-TH-05 (Holmarc Opto-Mechatronics Ltd, Kochi, India). 20 μl of ACM solution was placed at the center of the PCL-GO scaffold and rotator was spun at 300 rpm for 20 s. The ACM coated scaffolds were left for drying at room temperature in the laminar hood overnight and used for further characterization.

### Physicochemical characterization

#### Scanning electron microscopy (SEM) and energy dispersive x-ray spectroscopy (EDS)

Electrospun scaffold were gold coated and imaged using a FESEM equipped with EDS (JSM 7600 F, JEOL, Japan) under a 10-kV accelerating voltage. The surface morphology and the diameter of the electrospun nanofibers were analyzed using Image J software.

#### Raman spectroscopy

The electrospun scaffolds were subjected to Raman analysis to determine the presence of GO. Raman spectroscopy was conducted using a 20x objective on a Renishaw inVia Raman Microscope with an Ar laser (514 nm).

#### Mechanical strength analysis

Mechanical properties of neat PCL and PCL-GO nanofibrous scaffolds were determined using a Shimadzu universal texture analyzer (EZ-SX Shimadzu Corporation, Japan). The electrospun nanofibrous scaffolds were cut with scissors into 30 mm length and 5 mm width. Sample thickness and the gauge length were measured using a digital vernier calipers. The two ends of the nanofibrous scaffolds were clamped onto the instrument and stretching rate of 20 mm min^−1^ was applied till fracture. The stress and strain percent were calculated and the curve was plotted. Scaffolds were tested in triplicates.

#### Contact angle measurement

The contact angle of the nanofibrous scaffolds was measured using a goniometer (HO-IAD-CAM-01). The scaffold was placed on the goniometric stage on which a drop of water was gently placed, images were captured, and measurements were done using the instrument software.

#### DPPH assay

The neat PCL and PCL-GO solutions and their corresponding electrospun scaffolds were assessed for free radical scavenging potential by DPPH (2,2-diphenyl-1-picrylhydrazyl) assay. The PCL and PCL-GO solutions and their corresponding scaffolds were placed in a 24 well plate. 0.1 mM DPPH solution was prepared in ethanol and around 4 ml was added to the samples and allowed to react in dark for 20 min at room temperature. A DPPH blank without sample was used as control. The obtained colored solution was subjected to absorbance measurement at 517 nm using a multi-mode plate reader (HH34000000, Perkin Elmer (Ensight)). The radical scavenging activity was determined using Eq. 1.

1 $$Radical\,scavenging\,activity( \% )=\left(\frac{Ac-As}{Ac}\right)\ast 100$$

where Ac is the absorbance of control and As is the absorbance of the scaffold/solution.

#### Cytochemical staining

Cytochemical staining for various components of extracellular matrix was performed for quantitative analysis of ACM coating over neat PCL and PCL-GO scaffolds. To verify the extent of ACM coating over the scaffolds, ECM components like glycoproteins and collagen were assessed. To visualize glycoproteins, alcian blue staining was performed, for collagen, Mason’s trichome staining was performed and to assess proteins, commassie blue staining was carried out. Imaging was conducted using a fluorescence microscope (Nikon Eclipse- TE2000-U).

#### Cell viability and proliferation

A live/dead assay kit was used to evaluate viability of cells cultured on the scaffolds. Live/dead assay comprises of fluorescent dyes containing calcein-AM and ethidium bromide-1 which stain live cells green and dead cells red, respectively. The scaffolds were sterilized on each side by placing it under UV for 1 h. Scaffolds used for the analysis were neat PCL scaffolds, PCL scaffolds coated with ACM (PCL-ACM), PCL-GO scaffolds and PCL-GO scaffolds coated with ACM (PCL-GO-ACM). The scaffolds were placed in 24 well plates and complete media was added and incubated for 5 min at 37 °C in 5% CO_2_. Following which, approximately 10,000 RSC-96 cells were seeded, and culture media was changed every alternate day. On day 3, the calcein-AM and ethidium bromide-1 dyes in the ratio of 1:4 were diluted in 2 ml PBS and about 50 μl of the prepared dye solution was added to each well and plates were subsequently incubated in dark for 1 h at 37 °C. The well plates were observed under the fluorescence microscope (Nikon Eclipse- TE2000-U) for green (live) and red (dead) cells. The images were analyzed and percentage viability was calculated using the Image J software. Additionally, in order to quantify the cell viability and assess the metabolic activity of RSC-96 cells, MTT assay was carried out. Approximately 50,000 cells were seeded in their log phase to the scaffolds with complete media. After incubation for 1 and 4 days, MTT reagent was added to the well plates and incubated in dark for 3 h at 37 °C. Formazan crystals formed at the end of the incubation time were dissolved in DMSO and kept on a plate shaker for 30–45 min and absorbance was measured using a multi-mode plate reader (HH34000000) at 570 nm (*n* = 3).

#### Cell adhesion analysis

Cell adhesion was analyzed by evaluating the SEM images 8 h after cell seeding, as mentioned in previous studies [24]. Briefly, the cell seeded scaffolds were washed with PBS and fixed with 2.5% glutaraldehyde at 4 °C overnight. After incubation, the scaffolds were washed with PBS and dried and stored in vacuum desiccator for further analysis. The fixed scaffolds were gold coated and observed under FE-SEM (JSM 7600 F, JEOL, Japan).

#### DCFDA assay using flow cytometry

2′,7′-Dichlorodihydrofluorescein diacetate (DCFDA) is the most commonly used assay to quantify oxidative stress. DCFDA penetrate cell membrane and react with reactive oxygen species (ROS) and gets oxidized to form green fluorescing 2′,7′-dichlorofluorescein (DCF). A 10 mM DCFDA stock solution was prepared in methanol and diluted with the DMEM culture media to a concentration of 100 μM. 50,000 RSC-96 cells were seeded on the scaffolds in a 48-well plate and cultured for three days. After three days, cell seeded scaffolds were treated with 10 μM of H_2_O_2_ for 20 min. Subsequently, H_2_O_2_ exposed cells were washed once with PBS and 100 μM of the DCFDA solution was added for 30 min in dark at 37 °C in a humidified incubator. Stained cells were washed once with PBS, trypsinized, filtered and analyzed immediately in a BDLSR II flow cytometer, with excitation at 485–495 nm with corresponding emission at 520–530 nm. Scaffolds tested include TCPS (control), PCL scaffolds, PCL-ACM scaffolds, PCL-GO scaffolds, and PCL-GO-ACM scaffolds. All gain and amplifier settings were held constant for the duration of the experiment. BD FACSDiva software was used to acquire the data while the analyzes were performed on the De Novo FCS Express v6.0. Statistical representation of the plots was done using the GraphPad Prism (*n* = 2).

#### Gene expression analysis

The RSC-96 cells were cultured on the scaffolds for 7 and 14 days. Approximately 10,000 cells were seeded on the scaffolds in 6 well plates. At each time point, RNA isolation was carried out using the TRIzol method and reverse-transcribed to cDNA to check gene expression for peripheral myelin protein 22 (PMP22) gene in quantitative RT-PCR (QuantStudio 5 machine) for 100 cycles. The sequence of primers is listed in Table 1. The qRT-PCR cycle including denaturation, annealing, and extending phases occurred at 95 °C, 53 °C, and 94 °C for 30, 30, and 5 s, respectively. Quantitative expression of the gene was calculated after normalizing against the endogenous housekeeping gene GAPDH and the data was analyzed using the delta-delta CT method (*n* = 3).

**Table 1:** Primer sequences used for real-time polymerase chain reaction (RT-PCR)

PRIMER	FORWARD SEQUENCE	REVERSE SEQUENCE
GAPDH	GACATGCCGCCTGGAG AAAC	AGCCCAGGATGCCCTTT AGT
PMP22	AATAATCCGCTGCCCG AATCAAG	CTCCGCCTTCAGGGTCA AGTC

### Statistical analysis

All the quantitative experiments were conducted in triplicates and the data are presented as mean ± standard deviation. Statistical analyzes were performed using ANOVA test with *p* < 0.05, and *p* < 0.01 as a criterion for statistical significance using GraphPad Prism software.

## Results

### Scaffold morphology

**Fig. 3:**
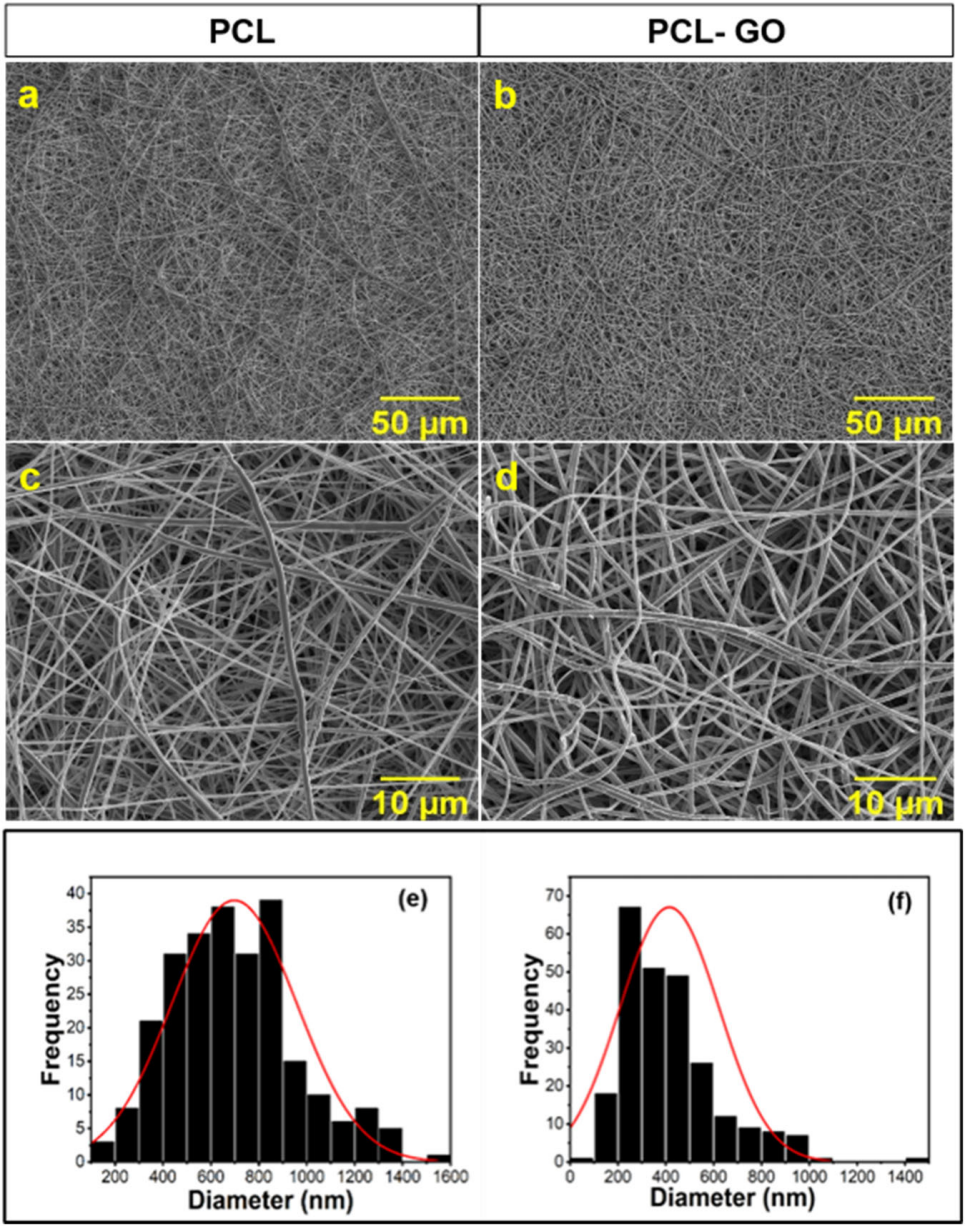
Scanning electron microscopy images of (**a**) and (**c**) PCL nanofibers; (**b**) and (**d**) PCL-GO nanofibers. **a**, **b** 1000x magnification, scale bar = 50 μm; **c**, **d** 5000x magnification, scale bar = 10 μm. Frequency distribution graph of the diameter of (**e**) PCL scaffolds (**f**) PCL-GO scaffolds. Number of fibers analyzed in each group is *n* = 250

Neat PCL and PCL-GO dispersions were electrospun to obtain nanofibrous scaffolds. SEM imaging of the scaffolds revealed random fiber morphology with the fiber diameter of 697.76 ± 260.37 nm and 415.47 ± 206.88 nm for neat PCL and PCL-GO scaffolds, respectively (Fig. 3). EDS analysis of the electrospun scaffolds revealed an increase in carbon (C) and oxygen (O) amounts in PCL-GO scaffold (C – 72.29 ± 0.46 wt%, O – 27.01 ± 0.3 wt%) compared to neat PCL scaffold (C – 18.86 ± 2.95 wt%, O – 3.64 ± 0.36 wt%). Next, we conducted contact angle measurement to analyze the change in surface charge of the electrospun scaffolds in regard to the inclusion of hydrophilic filler, GO in hydrophobic PCL matrix. Contact angle values reduced from ~133° on neat PCL to ~129° on PCL-GO scaffolds.

### Raman spectroscopy

**Fig. 4:**
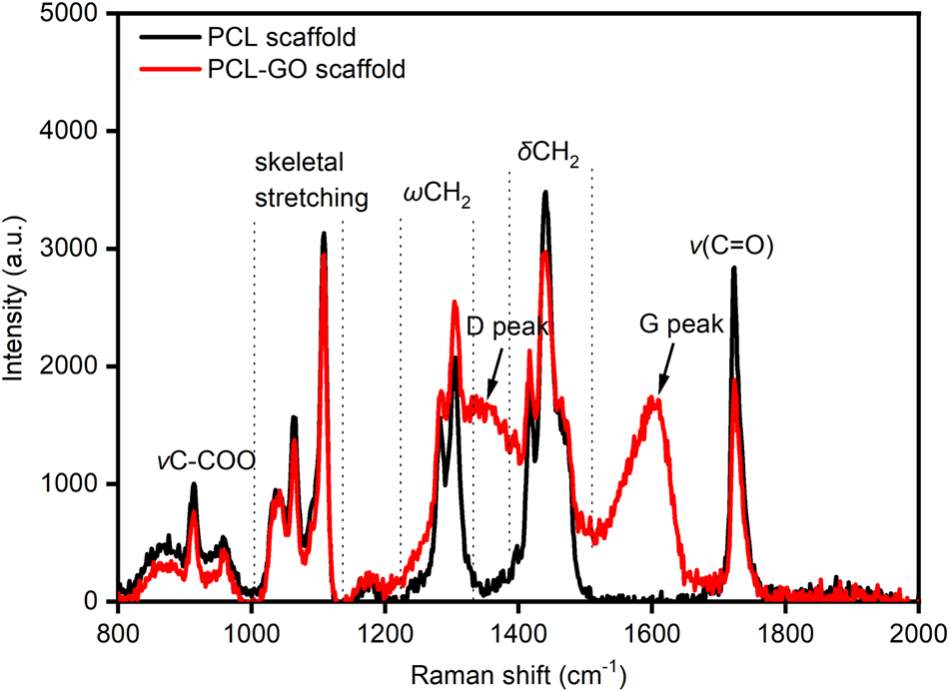
Raman spectra of electrospun PCL and PCL-GO scaffolds. The presence of D and G peak indicates the presence of GO in PCL-GO scaffolds

Raman analysis was conducted to confirm the presence of GO in the electrospun PCL-GO scaffold. As shown in Fig. 4, emergence of distinct characteristic D and G peaks (of GO) at approximately ~1350 and 1609 cm^–1^, respectively confirmed the presence of GO in the electrospun scaffold. The D peak in the Raman spectra is ascribed to the presence of structural defects in the carbon lattice on the basal plane of GO whereas G peak is attributed to the hexagonal sp^2^-hybridized carbon cluster in the lattice on GO basal plane [44–46]. We also observed the presence of characteristic PCL peaks in both neat PCL and PCL-GO scaffolds. For example, the region 817 to 1010 cm^–1^ originate from the skeletal modes of C-COO stretch of aliphatic polyesters (such as PCL). The region 1006 to 1143 cm^–1^ is assigned to C-C skeletal stretching mode in agreement with previous reports [47]. The region 1217 to 1355 cm^–1^ is assigned to CH_2_ twisting mode and 1367 to 1532 cm^–1^ is ascribed to CH_2_ bending mode from the alkyl chain segment, while the sharp peak at ~1723 cm^–1^ is assigned to C = O stretching mode from the ester group. The sharp features in Raman spectra are indicative of the semi-crystallinity state of PCL.

### Mechanical strength

**Fig. 5:**
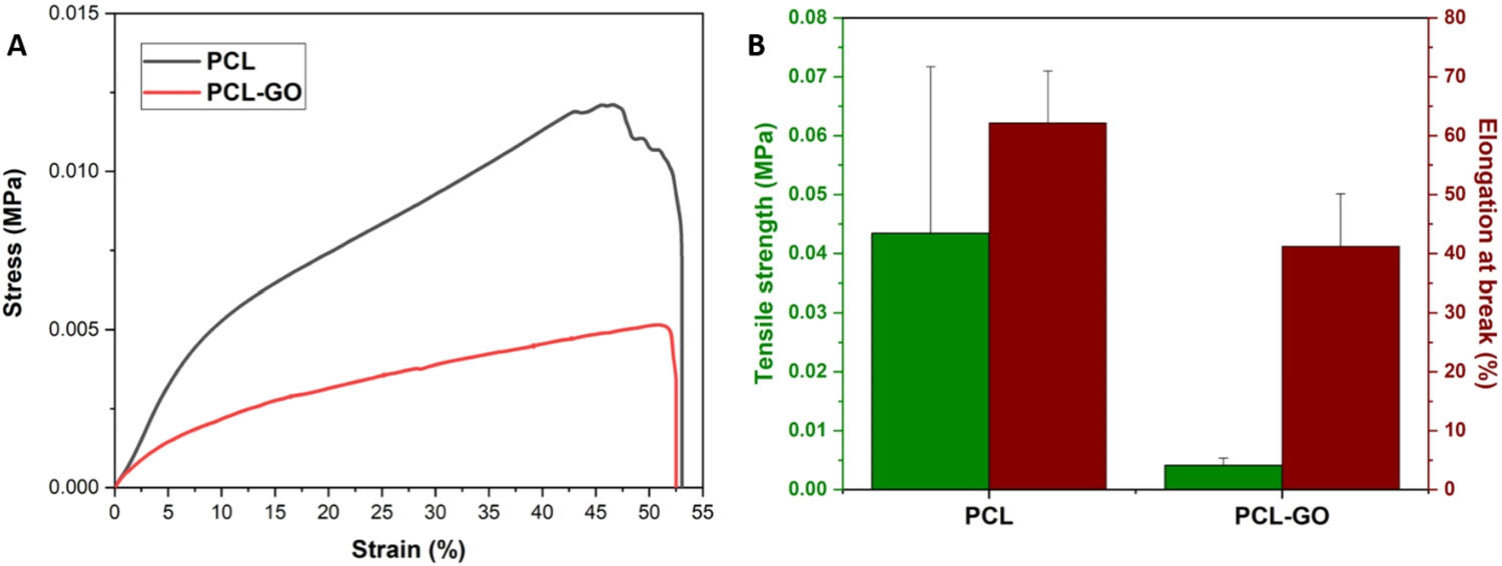
Mechanical properties of neat PCL and PCL-GO scaffolds. **A** Representative stress-strain curves, and (**B**) tensile strength and elongation at break. Data are presented as average ± standard deviation (*n* = 3)

Tensile testing was used to determine the mechanical properties of electrospun scaffolds (Fig. 5). The neat PCL scaffolds exhibited tensile strength of 0.043 ± 0.028 MPa, which reduced significantly by an order of magnitude with the inclusion of GO sheets in the polymer matrix (PCL-GO: 0.004 ± 0.002 MPa). Contrarily, there has been a marginal reduction in the stretchability of scaffold with the inclusion of GO sheets as adjudged from the change in the elongation at break (neat PCL: 62.14 ± 8.84%, PCL-GO: 41.20 ± 8.92%).

### DPPH assay

**Fig. 6:**
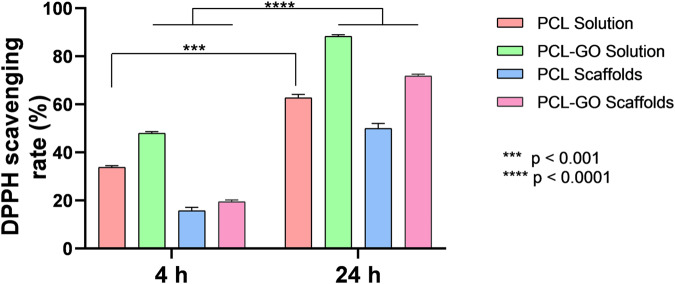
Free radical scavenging activity of PCL and PCL-GO solution and scaffolds analyzed by DPPH assay at 4 h and 24 h. Data are presented as average ± standard deviation (n = 2). Statistical analysis was performed with two-way analysis of variance (ANOVA) followed by Sidak’s multiple comparison test. ***p < 0.001 and ****p < 0.0001

To determine the antioxidant activity of GO in this study, we used a standard DPPH assay. Fig. 6 shows the DPPH activity of neat PCL and PCL-GO both in solution and in electrospun scaffolds. Both in solution and dry scaffolds, PCL-GO exhibited significantly higher DPPH activity compared to neat PCL regardless of the exposure time confirming the antioxidant ability of GO in these systems. The antioxidant performance was higher in the solution form for both neat PCL and PCL-GO compared to dried electrospun scaffolds.

### Cytochemical staining

**Fig. 7:**
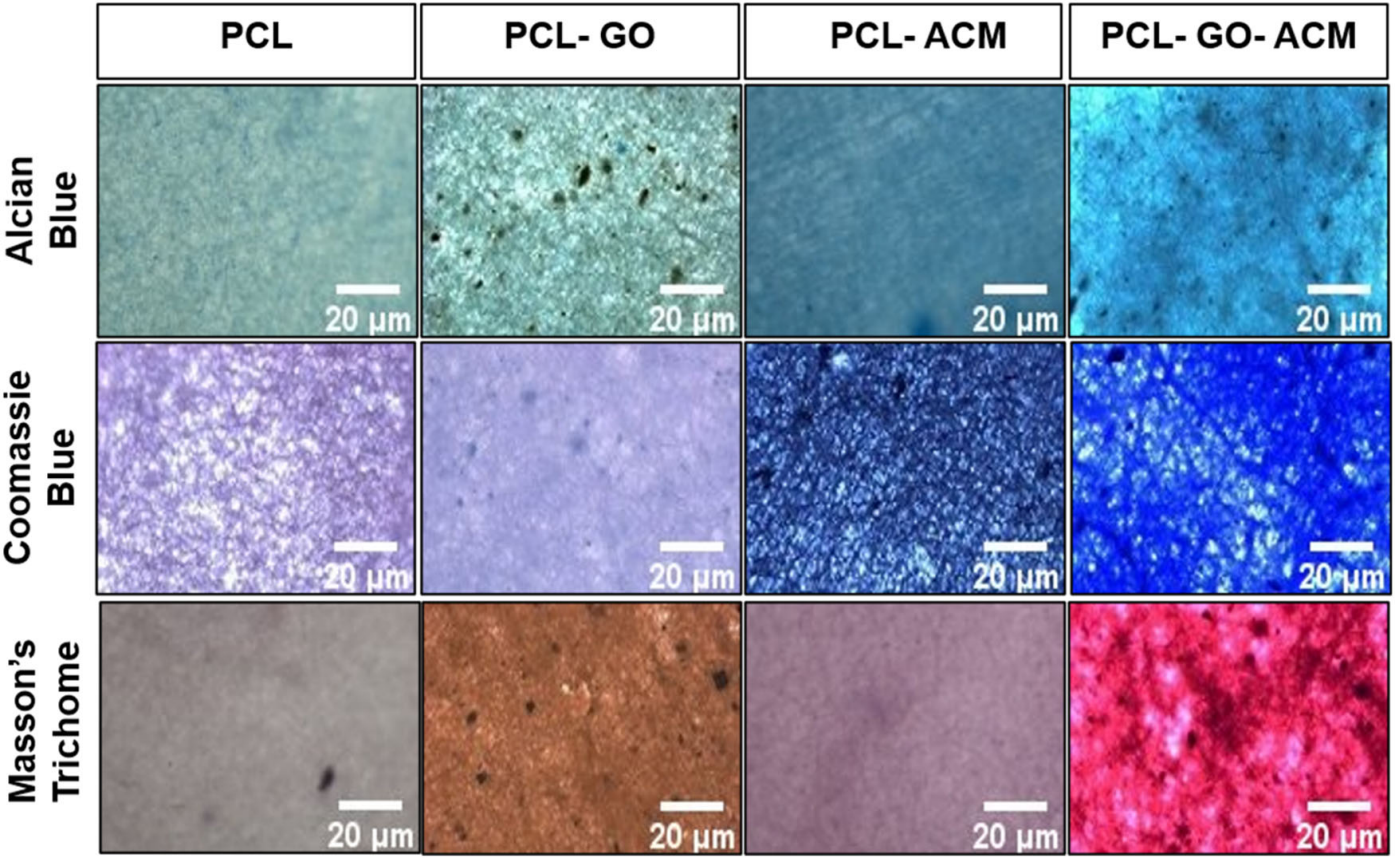
Cytochemical characterization of PCL-ACM and PCL-GO-ACM scaffolds. PCL scaffolds and PCL-GO scaffolds are taken as control. AB Alcian blue staining, CB Commassie blue staining, MT Masson’s trichrome stain. Scale bar = 20 µm

We performed cytochemical staining to determine the extent of ACM coating on neat PCL and PCL-GO scaffolds. Optical microscopy images of the coated scaffolds (PCL-ACM and PCL-GO-ACM) revealed a significant increase in the color intensity of all three stains indicating the presence of glycoproteins and collagen compared to their uncoated counterparts (PCL and PCL-GO). The three stains used were alcian blue for proteoglycans and glycosaminoglycans, coomassie blue for ECM proteins and Masson’s trichrome for connective tissue and collagen. Further, when comparing the two coated scaffolds, we also observed a stronger color intensity in PCL-GO-ACM scaffold compared to the PCL-ACM scaffold (Fig. 7).

### Cell proliferation and viability

**Fig. 8:**
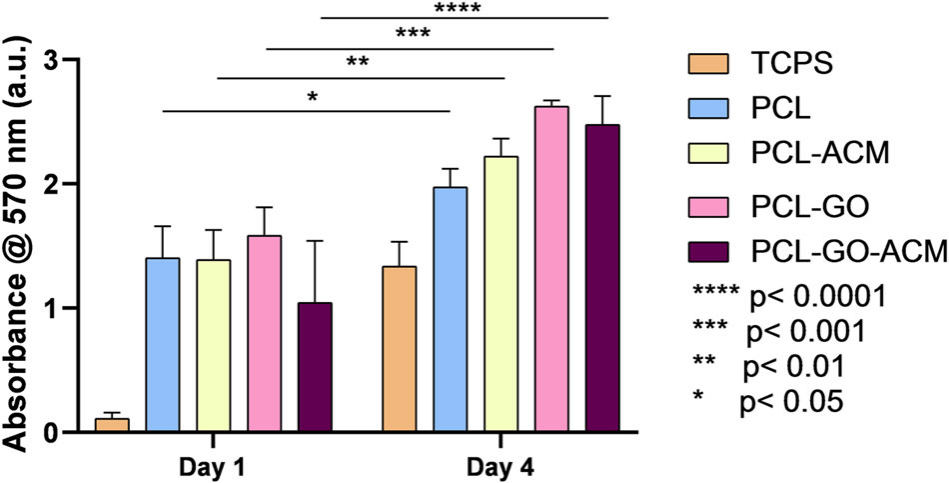
The bar graph represents the MTT assay performed at day 1 and 4 on PCL and PCL-GO scaffolds coated with or without ACM. All the scaffolds show an increase in proliferation when compared with day 1. TCPS served as control group. Data are presented as average ± standard deviation. Statistical analysis was performed with two-way analysis of variance (ANOVA) followed by Sidak’s multiple comparison test. *p < 0.05, **p < 0.01, ***p < 0.001 and ****p < 0.0001

The cytocompatibility of the electrospun scaffolds was conducted on RSC-96 cells using the standard MTT proliferation assay. PCL and PCL-GO scaffolds coated with or without ACM were studied. Cell proliferation study revealed significant increase in cell growth at day 4 after incubation compared to day 1 on all scaffolds including TCPS control indicating the biocompatibility of the scaffolds (Fig. 8). Further, PCL-GO scaffolds both with and without ACM coating (PCL-GO and PCL-GO-ACM) exhibited relatively higher cell proliferation than their neat PCL counterparts (PCL and PCL-ACM).

**Fig. 9:**
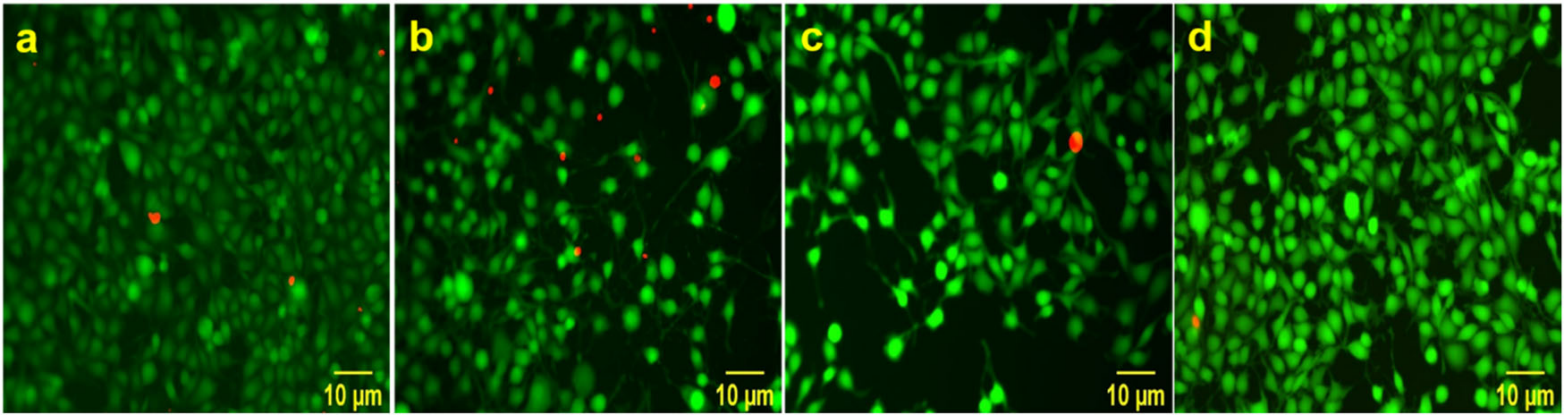
Representative images of Live dead staining for RSC-96 cells on scaffolds (**a**) neat PCL (**b**) PCL-ACM (**c**) PCL-GO (**d**) PCL-GO-ACM under a fluorescence microscope. The green fluorescence indicates live cells and red fluorescence indicates dead cells. Scale bar = 10 µm

**Fig. 10:**
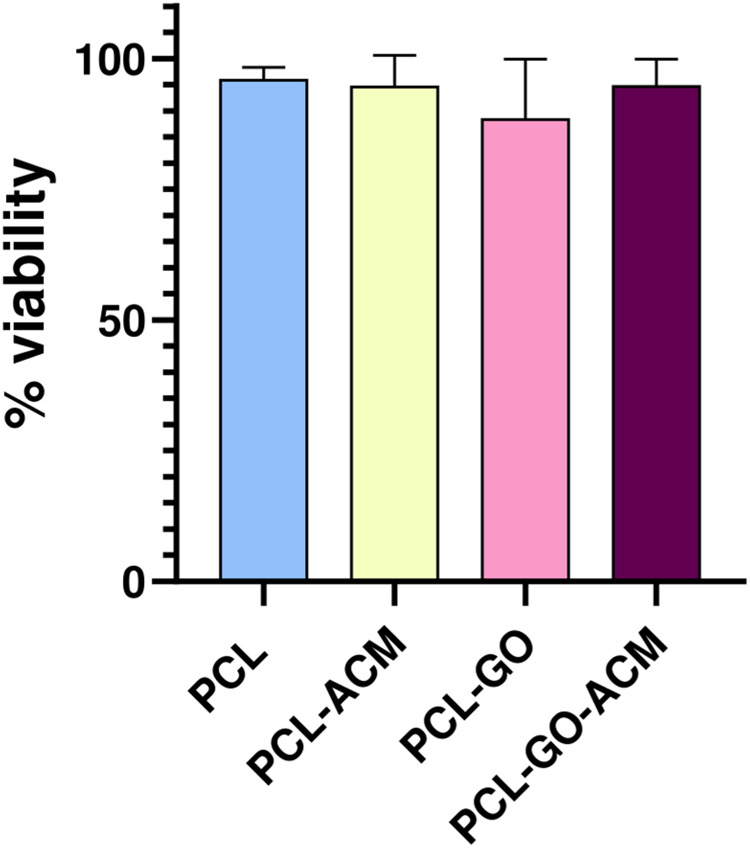
Graph represents percentage viability of RSC-96 cells at day 3. The cells were found to be viable in all analyzed samples ±. Data are presented as average ± standard deviation (*n* = 5)

Biocompatibility of both ACM coated and uncoated electrospun scaffolds was further assessed using the live/dead assay after incubating RSC-96 cells over 3 days on the scaffolds. Fig. 9 shows the presence of live (green) and dead (red) cells on electrospun scaffolds. Fluorescence images revealed significant cell viability as determined by the presence of predominant green cells in culture compared to dead cells on all scaffolds. Few dead (red) cells were observed in uncoated scaffolds (neat PCL and PCL-GO) compared to almost no dead cells observed on ACM-coated scaffolds (PCL-ACM and PCL-GO-ACM). We also observed the characteristic spreaded morphology on RSC-96 cells on all scaffolds, which was similar to the cell morphology observed in cell adhesion study under SEM. The graph of percentage cell viability as seen in Fig. 10 also further confirms the cellular compatibility of all the scaffolds.

### Cell-matrix interaction

**Fig. 11:**
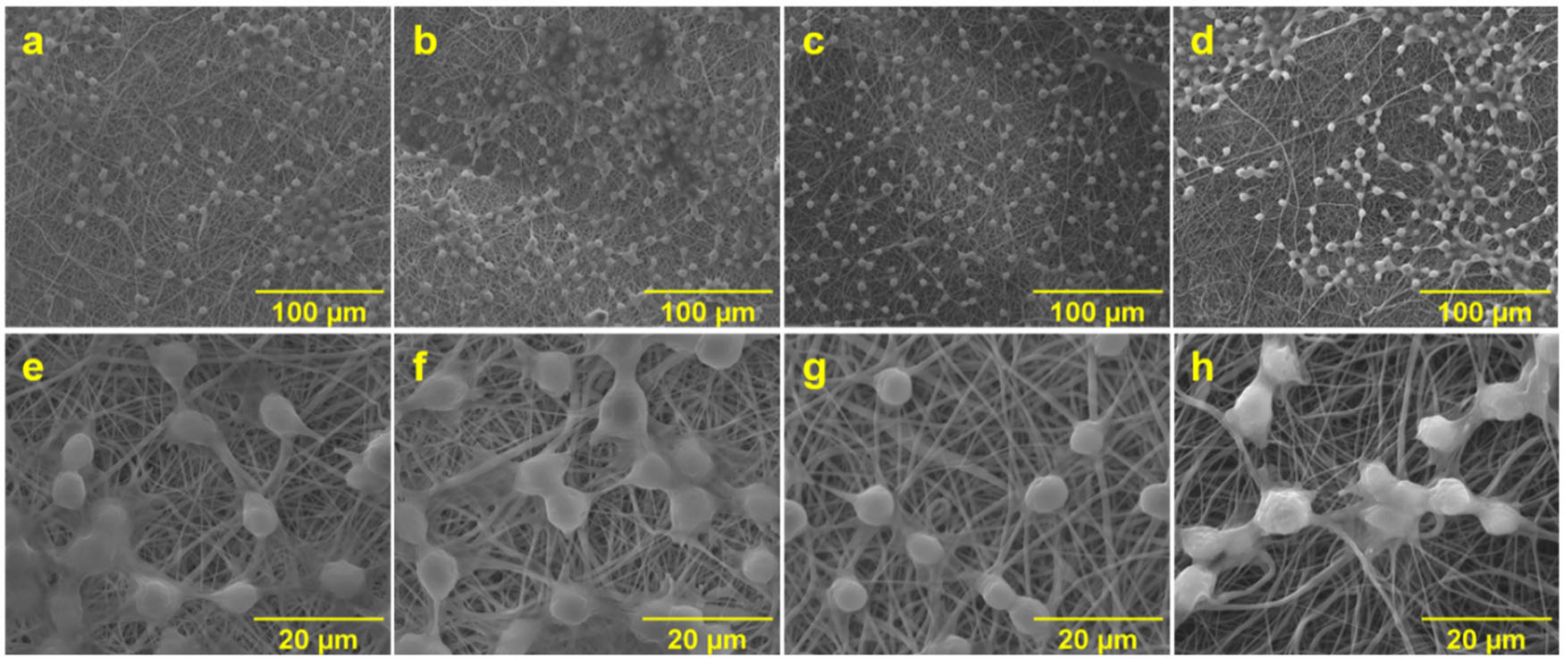
SEM image represents cell seeded scaffolds after 8 h incubation. **a**, **e** PCL scaffolds, (**b**, **f**) PCL-ACM scaffolds, (**c**, **g**) PCL-GO scaffolds, and (**d**, **h**) PCL-GO-ACM scaffolds. Top panel at 1000x magnification and scale bar = 100 μm. Bottom panel at 5000x magnification and scale bar = 20 μm

Cell-matrix interaction was evaluated using SEM analysis. Fig. 11 shows RSC-96 cell morphology on different scaffolds (coated and uncoated PCL and PCL-GO) over time. Images reveal cell adhesion as adjudged from extended cell morphology on all scaffolds at 8 h after incubation, this observation supports the assumption made regarding quicker cell adhesion on electrospun scaffolds. It appears that PCL-GO and PCL-GO-ACM scaffolds exhibit relatively higher cell area (extended cell morphology) compared to neat PCL and PCL-ACM scaffolds at all time points. No further change in cell morphology was observed with increasing incubation time (upto 8 h) on all scaffolds.

### DCFDA analysis

**Fig. 12:**
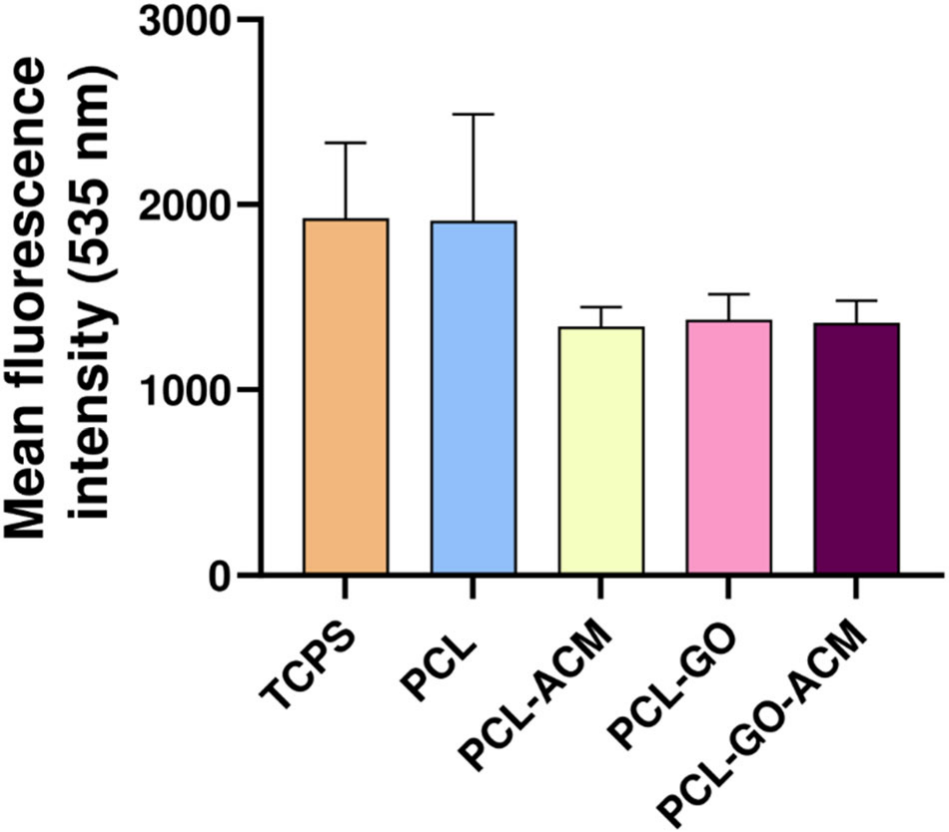
Quantitative analysis of antioxidant effect of nanofibrous PCL, PCL-ACM, PCL-GO, and PCL-GO-ACM scaffolds on RSC-96 cells by DCFDA analysis using flow cytometry. The TCPS served as control group. Data are represented as average ± standard error mean of two independent experiments

As shown in Fig. 12, the mean fluorescence intensity obtained from RSC-96 cells cultured on PCL-GO-ACM, PCL-GO, and PCL-ACM scaffolds was half-fold less than that of the RSC-96 cells cultured on PCL scaffolds and TCPS control.

### Gene expression analysis

**Fig. 13:**
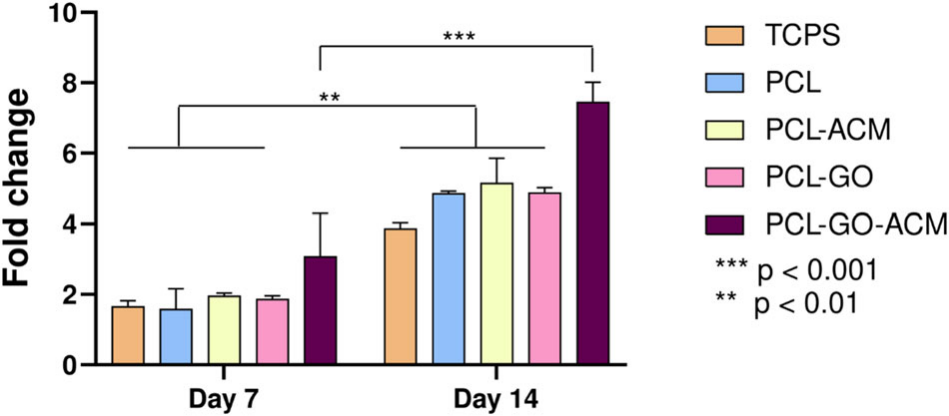
Comparative gene expression analysis of PMP22 gene by RSC 96 cells cultured on PCL, PCL-GO, PCL-ACM and PCL-GO-ACM scaffolds on day 7 and day 14. TCPS serves as control group. Data are represented as average ± standard error mean *(n* = 3). Statistical analysis was performed with two-way analysis of variance (ANOVA) followed by Sidak’s multiple comparison test **p < 0.01, ***p < 0.001

The influence of GO and ACM incorporation in PCL scaffolds on myelination was examined by using RT-qPCR by culturing RSC-96 cells on PCL, PCL-ACM, PCL-GO, and PCL-GO-ACM scaffolds for 14 days. As shown in Fig. 13, PMP22 gene expression increased significantly (*p* < 0.01) with time in all scaffolds. The extent of increase in gene expression (almost double) in PCL, PCL-ACM, and PCL-GO scaffolds was only marginally better than TCPS control which did not reach significance at day 14. In the case of PCL-GO-ACM scaffold, we observed a relative increase in PMP22 expression compared to other scaffolds (PCL, PCL-ACM, and PCL-GO) and control (TCPS) at day 7. At day 14, the increase in PMP22 gene expression was more significant in PCL-GO-ACM scaffolds (p < 0.001) from day 7 than other scaffolds (*p* < 0.01). At day 14, PCL-GO-ACM scaffold outperformed other scaffolds and control in PMP22 gene expression.

## Discussion

Schwann cells are principle glial cells in peripheral nerves originating from the neural crest. Schwann cells are also multipotent i.e. they can differentiate into other glial subtypes in the PNS. Schwann cells play a major role in peripheral demyelinating diseases as they help in remodeling and regenerating the damaged axons. When an axon is injured, the Schwann cells undergo dedifferentiation and help the macrophages to clear the myelin debris that forms as a result of the injury. Once the injury site is cleared, migration of Schwann cells to the injury site occurs to remyelinate the injured axons [2, 11, 48]. Current treatment strategies for peripheral demyelinating diseases include cell based therapeutic approaches. Even though these existing strategies demonstrate remyelination on acute demyelination, their potential in chronic conditions remains unexplored. Using tissue engineering approaches, a biomaterial can be developed for stimulating the non-functioning Schwann cells towards remyelination to offer long-term neuroprotection. In this study, we aim to fabricate a biodegradable biomaterial based on electrospun PCL nanofibers recapitulating the topological architecture of innate tissue matrix to support Schwann cell growth and their remyelination ability. Further, PCL was blended with GO to exploit its (GO) antioxidant property in terms of scavenging reactive oxygen species. To introduce biochemical cues, we used Schwann cell decellularized ECM (ACM). These multifunctional nanofibrous scaffolds were explored for their neuronal regeneration efficacy using Schwann cells.

The PCL and PCL-GO nanofibrous scaffolds were electrospun and were analyzed for their morphology using SEM analysis. We observed no significant increase in fiber diameter with the inclusion of GO from the SEM analysis, this observation was in line with previous reports of polymer/filler electrospun scaffolds [49]. The increase in both C and O atomic wt% in PCL-GO observed by EDS analysis was expected with the inclusion of GO which contains significant number of oxygen-containing functional groups. The observed reduction in contact angle was also in agreement with our hypothesis that hydrophilic GO will reduce the surface charge of an otherwise relatively hydrophobic neat PCL scaffold. To further analyze the scaffolds for their molecular structure, Raman spectroscopy was performed. The presence of the characteristic D and G peaks confirmed the presence of GO within the PCL-GO scaffolds, which was in agreement with previous reports [42–44]. Furthermore, the crystallinity of PCL was retained despite the inclusion of GO which was also confirmed by Raman spectroscopy. This was established by no observable change in the characteristic PCL peaks in the Raman spectrum of the PCL-GO scaffold highlighting that the inclusion of GO caused no structural or conformational change in the intrinsic crystallinity of PCL [50, 51]. Raman analysis validated an important requirement that an inclusion of a filler should not interference with the intrinsic properties of the polymer.

We performed mechanical strength analysis to observe the effect of the inclusion of GO in nanocomposite scaffolds. Tensile strength alludes to the maximum stress a scaffold can bear before failure and elongation at break is indicative of the maximum strain before failure. The reduction in tensile strength is somewhat unexpected. When compared to previous reports where tensile strength increases with an inclusion of a carbonaceous filler [52], the reduction observed in this study is postulated to have been caused by disruption to the organization of polymer chains during scaffold preparation with the inclusion of GO sheets compromising their (scaffold) ability to dissipate stress. Such disruption by filler particles also disrupts intrinsic movement of polymer chains and their organization within a scaffold which can lead to a reduction in the stretchability of the scaffolds as observed here. The observed reduction in elongation at break of polymer matrices (films, scaffolds) are in agreement with previously reported studies [41, 53–56].

In this study, GO was used as a potential antioxidant filler. Antioxidant fillers function by scavenging the free radicals from the solution and thereby prevent oxidative stress mediated damage particularly in biomedical applications [6]. As anticipated, antioxidant performance improved significantly albeit at similar rates with increasing exposure time regardless of the conditions (solution and electrospun scaffolds, both with and without GO). Such performance difference can be explained by relatively higher surface area exposed to react with DPPH in solution compared to only the two-dimensional surface (with significantly less surface area) exposed in electrospun scaffolds.

Based on our previous work where we showed that the Schwann cell-derived ACM can provide myelination cues, we expanded on this advantage in this study where ACM was spin coated over the neat PCL and PCL-GO nanofibrous scaffolds [24, 41]. To confirm the uniform coating of ACM, cytochemical staining was performed. Alcian blue staining was done to evaluate the presence of proteoglycans and glycosaminoglycans, Coomassie blue for ECM proteins and Masson’s trichrome for connective tissue and collagen. The intensity of different stains is expected to be higher in the ACM coated scaffolds (PCL-ACM and PCL-GO-ACM) due to the presence of different intrinsic ECM components (i.e. collagen, proteoglycans and glycosaminoglycans and other ECM proteins) compared to the uncoated samples (neat PCL). The pale color observed in uncoated controls in cytochemical staining may be caused by non-selective absorption of the dyes by the porous nanofibrous scaffolds. However, the significant increase in intensities was observed in the case of ACM coated scaffolds which were in agreement with previously published reports [24, 41, 57, 58]. The observed differences could be attributed to the potential interactions of ACM components with oxygen-containing functional groups of GO in the PCL-GO-ACM scaffold. It has been previously reported that oxygen-containing functional groups of GO can interact with primary amines (similar to the one present in ACM components) at room temperature – we postulate that this could be one of the reasons leading to improved ACM coating on the PCL-GO-ACM scaffold leading to higher color intensities [46, 59].

The biological activity of rat Schwann RSC96 cells such as cell viability, proliferation, and adhesion were then evaluated qualitatively and quantitatively using MTT and live-dead assays, respectively. The MTT assay exhibited significant increase in cell growth at day 4 in comparison to day 1 and PCL-GO and PCL-GO-ACM scaffolds exhibited higher cell proliferation than neat PCL and PCL-ACM scaffolds. The marginal difference in cell proliferation observed on day 1 in the MTT assay amongst different scaffolds could be due to the different number of cells surviving after 1 day on different scaffolds. The considerably higher values at day 4 on electrospun scaffolds (both ACM coated and uncoated) relative to TCPS is postulated to the combination of (i) increase in cell numbers due to cell growth, and (ii) potentially higher cell survival post seeding on the electrospun scaffolds. Electrospun scaffolds with nanofibrous morphology provide cell adhesion sites enabling quicker cell adhesion than flat TCPS surface. It is postulated that quicker cell adhesion will provide the environment for cells to start proliferating which could explain higher cell numbers in electrospun scaffolds compared to TCPS at both day 1 and day 4. The increase in cell proliferation on PCL-GO scaffolds (PCL-GO and PCL-GO-ACM) as seen in the MTT assay could be attributed to the (i) greater cell adhesion on these scaffolds due to the presence of GO sheets, and (ii) relatively higher surface polarity than neat PCL scaffolds (PCL and PCL-ACM). The role of surface polarity can also be reasoned for marginal increase in cell proliferation in ACM coated PCL scaffolds (PCL-ACM) compared to neat PCL scaffolds. Taken together cell proliferation and viability studies confirmed the cytocompatibility of both ACM coated and uncoated PCL and PCL-GO scaffolds.

During normal physiological functioning of our body, free radicals are inherently generated by mitochondria of virtually all cells [6]. These free radicals are scavenged by the mitochondrial defense mechanism and the endogenous antioxidants [6, 60]. Generally, when the reactive oxygen species (ROS) production is increased along with the decrease in activities of enzymes like superoxide dismutase (SOD), catalase and peroxidases cell death occurs [61, 62]. Similarly in demyelinating diseases, there is an increase in the production of free radicals which causes damage to the insulating myelin leading to cell death [61–64]. Therefore, we examined the effects of the scaffolds on ROS production using DCFDA staining by flow cytometry analysis. DCFDA staining showed that the fluorescence intensity of PCL-ACM, PCL-GO, and PCL-GO-ACM scaffolds was less than neat PCL scaffold indicating the greater radical scavenging activity of PCL-ACM, PCL-GO, and PCL-GO-ACM scaffolds. However, in our study, the presence of GO has not elevated the antioxidant property of the PCL-GO and PCL-GO-ACM scaffolds compared to their PCL and PCL-ACM counterparts. Our results are similar to a previous study, where ACM derived from human umbilical cord-derived mesenchymal stem cells (UCMSCs) showed significant reduction in ROS when compared to control confirming the potential antioxidant nature of the ACM [38]. In another study, Wharton’s Jelly mesenchymal stem cells (WJMSCs) derived ECM also provided resistance in cardiac c-kit cells from ROS mediated oxidative stress indicating the potential antioxidant nature of proteins found in the ECM [39].

PMP22 is one of the pro-myelinating factors exhibited by rat Schwann cells. In peripheral demyelinating diseases, PMP22 gene undergoes mutations causing disruption of myelin sheath and axonal structure leading to various disabilities [65]. Our results showed that there was significant increase in PMP22 expression on PCL-GO-ACM scaffolds from day 1 to day 14 and also when compared to other scaffolds, PCL-GO-ACM scaffolds showed increased PMP22 expression. We hypothesize that the significant outperformance of PCL-GO-ACM scaffold on PMP22 expression is indicative of a combinatorial effect of GO and ACM coating, and thus on myelination. This hypothesis is supported by the data showing no considerable change in PMP22 gene expression when each component GO (PCL-GO scaffold), and ACM coating (PCL-ACM scaffold) was examined in isolation. The impact of ECM proteins in ACM and the presence of laminins and laminin-binding integrins on myelination and behavior of Schwann cells have been well documented [66–70], which can explain the observed significant increase in PMP22 expression over time on ACM coated scaffolds. Shah et al. [71] studied PCL electrospun scaffolds surface coated with GO for neural stem cell (NSC) differentiation into oligodendrocytes, which are primary glial cell-types of CNS responsible for myelination of axons. It was observed that there was a significant increase in the expression of a mature myelination marker; myelin basic protein (MBP) in PCL/GO electrospun scaffolds compared to uncoated PCL electrospun scaffolds as well as GO coated on a glass substrate. Thus the synergistic effect of PCL and GO as a combination to improve the myelination was established in their study [71]. However, the need for surface coating with laminin to promote NSC adhesion on PCL/GO scaffolds was also emphasized [71]. In our study, ACM was in itself fulfilling the role of laminin in terms of promoting the cell adhesion and eventually on PMP22 gene expression.

Thus, in this study, we systematically investigated how electrospun PCL and GO-based nanofibrous scaffolds coated with ACM regulate oxidative stress and myelination in Schwann cells. Our study clearly indicates that the fabricated scaffolds hold the great potential for application in nerve regeneration and treating demyelinating disorders.

## Conclusion

In this study, multimodal PCL-GO nanofibrous scaffolds were fabricated using electrospinning and subsequently coated with Schwann cell derived acellular matrix (ACM). In these scaffolds, GO was used as a potential antioxidant and ACM was utilized for its microenvironment and biochemical cues leading to improved cell adhesion, proliferation and remyelination. Incorporation of GO led to no considerable change in nanofiber morphology or size. The presence of GO was confirmed by Raman analysis. The antioxidant activity of GO in PCL-GO scaffolds was confirmed by DPPH assay, while the uniformity of ACM coating on the scaffolds was confirmed by cytochemical staining. In vitro studies using Schwann RSC96 cells, revealed significantly increase in cell proliferation in combinatorial scaffold (PCL-GO-ACM) over 4 days. PCL-GO-ACM promoted significant remyelination as adjudged from the PMP22 gene expression in RSC96 cells over 14 days compared to control and scaffolds comprising one of the two components. This study highlights that combination of antioxidant and intrinsic ACM coating in bioscaffolds are required to promote remyelination. The outcome of this work is expected to promote further investigation in the field and lead to potential development of a clinical modality for peripheral nerve regeneration.
